# The *REAL* journey: a meaning-oriented approach to moral injury care

**DOI:** 10.3389/fpsyt.2026.1774486

**Published:** 2026-03-31

**Authors:** Melissa A. Smigelsky

**Affiliations:** 1 Integrative Mental Health, U.S. Department of Veterans Affairs, Durham, NC, United States; 2 Department of Psychiatry, University of North Carolina Chapel Hill, Chapel Hill, NC, United States

**Keywords:** constructive psychotherapy, group psychotherapy, logotherapy, meaning reconstruction, moral injury

## Abstract

This paper describes three theoretical models that focus on meaning-making as a foundation for moral injury conceptualization and care, as manifested in the *Reclaiming Experiences and Loss (REAL)* moral injury group therapy. The philosophical foundation of the *REAL* group is Viktor Frankl’s logotherapy, which emphasizes the role of the human spirit and posits that meaning is achieved through living out one’s fundamental responsibility to actualize whatever values are available in a given moment. Constructive psychotherapy operates from core assumptions about client autonomy, an inherent drive toward meaning, the influential impact of experiences with others, and a capacity to live within one understanding of reality while simultaneously considering other possibilities. These assumptions shape the format and processes of the *REAL* group. Finally, meaning reconstruction provides the overarching structure for processing grief and loss that retains sufficient coherence around shared tasks and experiences to facilitate a group experience, while also maintaining space for each person’s particular journey of lamenting and reckoning with the aftermath of a moral violation. The *REAL* group therapy model promotes healing by emphasizing each person’s unique and particular opportunities for meaning while underscoring the common, shared human capacities and responsibilities that are fundamental to human existence.

## 1 Introduction

*“People experience life through frameworks of meaning that they create for themselves – both singly and collectively.” – Jonathan Raskin*

The term “moral injury” was introduced into the psychological literature to refer to a clinical syndrome arising in the aftermath of exposure to a “potentially morally injurious event” (1). Despite widespread resonance of the construct, there is not a single, unified definition or understanding of moral injury. The goal of this research topic on moral injury conceptualization is to “enhance understanding of how theoretical models can predict and influence the behaviors sustaining moral injury and influence treatment outcomes.” This begs a fundamental question of the extent to which any psychological theory on its own can reliably predict or influence behavior, given how complex and variable human behavior can be. Thus, theoretical models that implicitly or explicitly integrate diverse perspectives may hold particular promise. The contextual model of psychotherapy (2) is one example. It posits that there are three pathways by which psychotherapy leads to desired outcomes: 1) the real relationship, 2) expectations, and 3) specific ingredients. Specific ingredients comprise the part of the equation that is mostly closely linked to the theoretical model of understanding the problem and its remedy. This perspective paper will focus on meaning-making as the specific ingredient theorized to drive outcomes in the *Reclaiming Experiences and Loss* (REAL) moral injury group therapy.

“Meaning” has been defined as a *connection* between things (3). Thus, meaning-making can be understood as a process of seeking to connect things. In the aftermath of highly stressful life experiences, including morally injurious experiences, the desire to restore a sense of meaning is important (4). While research is limited, difficulties with meaning-making have been theorized and shown to contribute to worse mental health outcomes and spiritual struggles for veterans (5–8). Meaning-making is complex; it involves unconscious and deliberate processes, cognitive reappraisal, questions about significance, and a range of potential outcomes that include having made sense of what occurred, acceptance, reattributions of cause and effect, and changes to identity (4). Clearly, it would be challenging to operationalize these multifaceted components into a set of interventions that could be consistently implemented to reliably produce a desirable outcome. The subjectivity of meaning may actually render the task impossible. Yet the suffering of moral injury beckons us to try, and the *REAL* moral injury group offers one attempt.

In the *REAL* group, meaning-making in the aftermath of a moral violation is understood and approached through three complementary theories. The philosophical foundation is Viktor Frankl’s logotherapy, which shapes two core assumptions that inform *REAL*: 1) the human spirit, which is distinct from the human mind, must be engaged to move beyond intellectual or cognitive processing and into deeper, existential meaning; and 2) healing from moral injury requires reckoning with, rather than seeking to ameliorate, the inevitability of suffering. Logotherapy asserts that finding or creating meaning in one’s life is an utterly unique and singular task (9) and thus requires intellectual and experiential humility on the part of the therapist/helper. This intellectual and experiential humility is embodied in a constructive approach to psychotherapy (10). Rather than adopting the posture of the expert with a mechanistic understanding of how to bring about change, the practical foundations of the *REAL* group includes striving to facilitate – but not direct – the therapeutic journey. Constructive psychotherapy does not progress toward a pre-determined desirable outcome (e.g., symptom reduction or behavioral change) but rather encourages the person seeking care to define the problem and solution in their own way. To facilitate exploration in a group setting, the theory of meaning reconstruction in the aftermath of loss (11) provides an orienting structure. By exploring loss in a non-pathologizing and deliberate way, a group of individuals can retain enough coherence around shared tasks to facilitate a meaningful journey together, while maintaining the particularity of each person’s path.

This paper describes how meaning-making is approached philosophically, practically, and structurally in the *REAL* moral injury group therapy. Definitions of moral injury vary, though they generally coalesce around common themes. In the *REAL* group, moral injury is understood to involve a violation of a fundamental moral belief or value. If the violation can be sufficiently explained or justified through contextualization and rational thinking, then it has not penetrated deeply enough into a person’s psyche to become moral injury. If the violation has become embedded, the lived experience of moral injury is characterized by a sense of stuckness – a seemingly insurmountable difficulty regaining one’s sense of footing in the world and living in a meaningful way. The particularity of the stuckness is directly determined by the uniqueness and singularity of the person’s existence. Recovery from moral injury thus necessitates willing experimentation with ideas, stories, and behaviors that allow for the construction of a revised world of meaning.

## 2 REAL group overview

The *REAL* group approach was developed by an interdisciplinary team of Veterans Affairs (VA) mental health and spiritual care providers in the United States, and it has been iteratively refined since 2015 (e.g., 12, 13). It has been implemented in more than a dozen VA facilities. *REAL* has evolved organically in response to feedback from group facilitators and veteran participants, particularly through text revisions and expansions of content to more fully reflect the range of morally injurious experiences reported by group participants (e.g., combat trauma, military sexual trauma, childhood abuse and neglect, perpetration of sexual assault, betrayal by trusted institutions) and the nature of suffering endured as a result. *REAL* consists of 13 sessions, which are 90 minutes long and take place either in-person or virtually, but not both (hybrid groups present logistical limitations that interfere with the therapeutic process). The sessions are co-facilitated by a trained team comprised of a mental health professional and a chaplain. *REAL* facilitators adopt a posture of *companioning* (14), which entails being present to pain, listening with the heart, honoring the spirit, respecting disorder and confusion, bearing witness, walking alongside, and engaging with compassionate curiosity (15).

The group structure is organized into three phases: 1) Exploration, 2) Lamenting Losses, and 3) Living into a New Life. Each phase culminates in a therapeutic activity that reflects the major tasks of that phase. The first phase culminates in making a commitment (by signing a companioning contract) to go on the journey together, showing up for oneself and each other. The second phase culminates in carefully curated sharing and bearing witness to stories of morally injurious experiences and the losses endured as a result. The third phase culminates in sharing and bearing witness to “new eyes” stories that integrate the morally injurious experience into the story and that contain a unique and particular vision for the future. These tasks were selected for their potential to carry deep and varied meaning for different group participants. More details about the *REAL* group can be found elsewhere (see 16, 17).

## 3 Theoretical models

In the following sections, each theoretical model that informs REAL will be presented with examples from the material that demonstrate its application.

### 3.1 Logotherapy

Moral injury has consistently been theorized to contain both psychosocial and spiritual dimensions, and empirical research has demonstrated those associations (e.g., 18–20). While it is possible to approach moral injury conceptualization and care from an exclusive lens (be it psychological, spiritual, relational, or even sociopolitical or environmental), doing so necessarily means neglecting some key aspect of what moral injury is about. The *REAL* group engages in a psychosocial-spiritual process by integrating the core psychological, spiritual, and relational dimensions of moral injury.

According to Viktor Frankl, logotherapy is “a psychotherapy which not only recognizes man’s [sic] spirit, but actually starts from it” (9, p. xvii). In particular, the human spirit, in Frankl’s view, is driven by a *will to meaning*, which is rooted in a fundamental consciousness and responsibility. Meaning is achieved through living out one’s fundamental responsibility, which is to actualize whatever values are available to the person in the moment. Frankl describes three types of values, actualized in different ways. Creative values are actualized through action, experiential values through appreciation, and attitudinal values through how a person endures an “unalterable fate” (9, p. 44). The opportunities available to any person in any particular moment are utterly unique and singular. No other person will ever face the exact situation in the exact same way. Similarly, the repertoire of options available to a person in a given moment is utterly unique and singular, such that someone else seemingly in the exact same situation would have unique and singular options available to them.

This perspective is particularly helpful when exploring the terrain of moral violations in the *REAL* group. It helps explain why an experience might be morally injurious to one person and not another, not only for whatever outward options were available at the time (context) but also because of the inner repertoire of utterly unique meanings/construings each person possessed. It also imbues the suffering of moral injury, the lamentation that flows out of recognition that things have gone terribly wrong, with spiritual significance and unique responsibility. Moral injury imposes something unalterable upon an individual. Individuals seeking help for moral injury may have all three categories of values available to them. They may actualize creative values and do something that restores justice. They may actualize experiential values through appreciation of loved ones, limbs, or landscapes. If ultimately no other options are available to them, they may actualize attitudinal values in the way they accept and bear seemingly unbearable suffering, with courage and dignity. Even self-condemnation, which is often regarded as something undesirable that should be eliminated or ameliorated in psychotherapy, can contain meaning in the form of an attitudinal value: “A man’s [sic] moral self-condemnation assumes an ideal of personality, his [sic] private ought-to-be. Thus, the man who judges himself harshly has caught sight of a value and is taking part in the world of values” (9, p. 57). What good is a helping professional who extracts a suffering person from the world of values?

The influence of logotherapy is expressed most directly in the *REAL* group through the subtle yet persistent emphasis on personal responsibility as a source of meaning. From the very beginning of the group, participants are assumed to have within them the wisdom they need to get unstuck from moral injury and to live into the unique and specific meaning of their lives. In session 1, group members are asked to imagine they are at the end of the group and provide an answer about what was useful about the group, what they needed and received/achieved. Whether or not they have words to express their knowing, it is assumed and impressed upon them that they possess the capacities they need. Similarly, part of the significance of the companioning contract in session 2 -- in which group members commit to journeying together -- is the acknowledgement that group members need each other, which implies that they each have something to offer. For the person who believes that they no longer possess a shred of goodness, that all capacity for redemption, vulnerability, or love is lost, no amount of skilled questioning or encouragement could convince them otherwise. They must live into that truth, through actualizing values in community.

### 3.2 Constructive psychotherapy

There is not yet consensus among experts about how to define and measure moral injury (21). This presents a conundrum for those who measure “success” of intervention according to changes in endorsement of externally defined symptoms or criteria. In his early moral injury work with Vietnam veterans, Jonathan Shay observed that some of them “achieved lives of great value to others and satisfaction for themselves” despite remaining “highly symptomatic” by DSM standards (22, p. 186). Relatedly, logotherapy views spiritual struggle as an accomplishment, rather than a symptom (9). What, then, is the purpose and goal of intervention, if so-called symptom reduction is neither practical nor particularly meaningful? Perhaps it is not to disrupt the processes maintaining moral injury but rather to create space for moral injury to be regarded as an unalterable experience of suffering that is, by its very nature, meaningful because it is an opportunity for actualization of values (9). Constructive psychotherapy offers an understanding of actualization (behavior), not as a dependent variable (i.e., an outcome which can be conditioned and thus predicted) but rather as an independent variable (10) – a way of asking a question that reflects both intention (rather than reaction) and potential for spontaneous (rather than conditioned) adaptation in each unfolding moment. This approach can be exercised with moral injury, which is without a codified problem definition or set of symptoms or processes to target, because the meaning of each action is personally derived.

Constructive psychotherapy shares features with postmodern theories and approaches that emphasize language, social context, and personal meaning (e.g., narrative, feminist, and emotion-focused therapies; 23) as well as modern cognitive-behavioral therapies such as Acceptance and Commitment Therapy (ACT) that exhibit a pragmatic focus on the context and function of behaviors (i.e., their viability as opposed to validity; 10) and that de-emphasize symptom reduction as a therapeutic goal in favor of improved functioning. However, constructive psychotherapists differ from their more traditional cognitive colleagues in the way they regard the relationship between the world as it “is” and the world as it is interpreted. While cognitive therapists seek to align a patient’s thoughts with “reality” as the therapist understands it, through interventions such as Socratic questioning and identification of cognitive distortions with the goal of reducing associated emotional distress, constructive therapists employ a broader interpretation of construing the world that includes rational thinking as well as feelings and body sensations (10), such that “reality” is comprised of all those elements. Furthermore, constructive therapists are committed to an egalitarian capacity for knowing, such that they do not have “better access to reality than their clients do” (10, p. 19). Constructive psychotherapies take an aggressively person-centered stance that refuses to situate the problem in objective terms but instead relies on idiographic experience to define the problem. This perspective is well suited to a challenge like moral injury, the experience of which is based on the subjective moral evaluation of what has occurred (24). At present, there are no agreed upon criteria to guide expert determination of whether a person is suffering from moral injury. Indeed, the individual alone has the power to say whether an experience was morally injurious, and whether the manifestation of their distress, whatever it may be, is the direct result of a moral violation. This is not a problem for constructive therapists, as personal constructs form the basis for understanding how an individual construes events.

Constructive psychotherapists utilize a variety of techniques and approaches and adhere to a general willingness to do whatever works in pursuit of “transforming client meaning-making” (10, p. 42). Nevertheless, there are four premises that guide a constructive perspective on people: 1) people are informationally closed systems; 2) people are active meaning-makers; 3) people are social beings; and 4) people are both ontological and epistemological construers (10). Important clinical implications are thus: 1) therapists cannot directly change people through intervention but rather must seek to stimulate the system to reorganize itself; 2) people are inherently driven toward meaningful construal of their experience of themselves and the world, and as such are inherently self-determining; 3) ongoing reorganization of the self-system is influenced (indirectly) by experiences with others; and 4) people operate within their constructs (i.e., their understandings of the world) while maintaining a capacity to question those constructs.

These constructive premises show up immediately in REAL through deconstruction of language, which is encouraged from the first session. Group members are explicitly informed that they will frequently hear the question, “What do you mean when you say (blank)?”, which invites illumination over assumption. Additionally, particular language (e.g., a definition of spirituality) is presented and group members are invited to react and respond to that language, potentially reconstruing individual and group understandings in the moment. This is in service of Premise 2 (i.e., that people are active meaning-makers), which encourages active rather than passive meaning-making. Stories and folklore are presented in the group to elicit potential self-organization by providing characters with which group members may strongly identify or against which strong contrasts may be drawn (25). These comparisons and contrasts may be more readily accessed indirectly through fictional characters (Premise 1). Similarly, the process group format provides opportunities for social interactions that may reinforce viable construals or inspire re-organization. Finally, group-based discussions and written assignments balance both reports of “reality” as the person understands it, and the safety that those construals provide, with invitations to consider other possibilities, without any pressure to adopt them (Premise 4).

### 3.3 Meaning reconstruction

Bereavement scholarship offers a model of meaning reconstruction in the aftermath of loss (11) that provides the overarching structure, and certain aspects of the contents, of the *REAL* group curriculum. To facilitate a meaningful group experience, it is important to have sufficient structure to retain coherence around shared tasks and experiences, while also maintaining space for each person’s particular journey. Morally injurious experiences involve losses of many kinds - from death losses (whether through perpetrating, failing to prevent, witnessing, or learning about them) to identity losses (e.g., innocence, capacity for social trust, ability to be a good fill-in-the-blank), to spiritual losses (such as one’s connection to a higher power or sense of meaning/purpose/significance). Thus, an intervention approach that draws from bereavement research and clinical practice is appropriate. In virtually all circumstances, moral injury entails a loss of “footing in the world” (26, p: 338). When “fragile expectations, understandings, and illusions meet with incompatible yet incontrovertible occurrences … at such moments, we can feel cast into a world that is alien, unimaginable, and uninhabitable, one that radically shakes or severs those taken-for-granted ‘realities’ in which we are rooted, and on which we rely for a sense of secure purpose and connection” (27, p. 10). This loss of meaning is fundamental to the experience of moral injury.

It takes time to recognize and identify moral injury-related losses, which might be part of the reason why some people suffer silently for decades after a moral violation. Some losses manifest right away, while others reveal themselves over time, perhaps through repeated perceived failures to live up to one’s moral standards. The experience of moral injury can be understood as a lament, a recognition that “relationships and circumstances have gone terribly wrong” (28, p. 110). This deep pain calls out for a process that can “transform unbearable pain into livable disappointment” (K. Meador, personal communication, May 7, 2021). Regarding moral injury as lament invites those who experience it to process it – intellectually, emotionally, and even physically in the context of a therapeutic group setting – through active grief. Moral injury is a type of traumatic experience that shatters fundamental assumptions that previously provided a connecting framework for making sense of oneself and the world (29). Naming and grieving what has been lost helps to separate the embers from the ashes, the opportunities and responsibilities that still hold potential from the stories and lies that ought to be stamped out. The goal of care, then, becomes reoxygenation of the embers.

In the *REAL* group, participants are guided through content, exercises, and discussions that help them identify losses they have endured related to their sense of self, their relationships (past and present), and their connection to whatever they believe to be sacred. They are introduced to concepts such as ambiguous loss (30) and disenfranchised grief (31). The goals of therapy with the bereaved form the roadmap for navigating moral injury-related losses: a) find a way to situate the *event story* (or morally injurious experience) within the broader story of the person’s life in a meaningful way; b) review the *back story* of the person’s relationship with the deceased (or moral formation) and how the event challenged that relationship (formation); and c) reclaim the person’s identity coherence, capacity for social trust, and/or connection to their deeply held beliefs/values (32). In REAL, this is done across multiple sessions focused on inventorying losses, followed by sharing and bearing witness to stories of loss (which is itself an act of remembrance), and finally several sessions devoted to intentional integration of the experience by building a connection between what was lost and what the person wishes to reclaim, restore, or create anew. These sessions are where the reoxygenation of the embers of meaning occur and are hopefully fanned into flames that provide a sense of security and comfort, and a renewed sense of footing in the world.

## 4 Conclusion

*“Love is the bridge between you and everything.” – quote attributed to 13^th^ century Sufi poet Rumi*

Participation in *REAL* entails personal and collective reflection on common existential themes that permeate human experience as well as personal and collective action geared toward actualizing the profoundly unique and specific opportunities for meaning in each person’s life. One theme those varied meanings often share is love. For one combat-exposed veteran, it was holding space for ongoing struggle with depression and anger while discovering that he could “love again.” For another, who experienced military sexual trauma, it was surrendering to “love of self” so that “new life for me can now begin.” Increasing one’s capacity for love is rarely a stated therapeutic goal; yet if love is truly the bridge between us and everything, then it stands to reason that in the pursuit of meaning in the aftermath of a moral violation, all roads lead to love. Those roads might lead to deeper recognition of who or what one loves (i.e., country, service, a specific person, or a sense of oneself), reimagined ideas of how to express one’s love (based on the actualization of values available to a person in a given moment), or living with the pain of what one was willing to sacrifice in the name of love (e.g., innocence, safety) which is made possible through reconstructing a world of meaning.

Each session of the *REAL* group concludes with the ritualized reading of a closing quote that captures some aspect of that session’s core idea. It is one of numerous small rituals built into the curriculum. Rituals provide “individual and communal opportunities for reasserting our personal and communal trust in a social and cosmic order” (30, p. 114). Moral injury destroys capacity for trust, in oneself and others, and thus reasserting trust becomes a necessary and intentional act. The final quote in the last session reminds participants that any ordinary action can be imbued with meaning, serving as a final reminder that meaning is a personal responsibility. The quote is attributed to writer Christina Baldwin and says, “Ritual is the act of sanctifying action – even ordinary action – so that it has meaning. I can light a candle because I need the light or because the candle represents the light I need.”

## Data Availability

The original contributions presented in the study are included in the article/supplementary material. Further inquiries can be directed to the corresponding author.
